# A189 IMPACT OF PHYSICAL ACTIVITY AND MEDICATIONS ON BODY COMPOSITION DYNAMICS IN PEDIATRIC IBD PATIENTS: A PROSPECTIVE STUDY

**DOI:** 10.1093/jcag/gwae059.189

**Published:** 2025-02-10

**Authors:** L Djani, K Orius, N Neale, L Dehbidi Assadzadeh, G Tongue, F Huang, X Yang, J Bouthot, C Deslandres, P Jantchou

**Affiliations:** Universite de Montreal Faculte de Medecine, Montreal, QC, Canada; Universite Laval Faculte de Medecine, Quebec, QC, Canada; Universite de Montreal Faculte de Medecine, Montreal, QC, Canada; Centre Hospitalier Universitaire Sainte-Justine, Montreal, QC, Canada; Ottawa University, Ottawa, KS; Universite de Montreal Faculte de Medecine, Montreal, QC, Canada; Universite de Montreal Faculte de Medecine, Montreal, QC, Canada; Universite de Montreal Faculte de Medecine, Montreal, QC, Canada; Centre Hospitalier Universitaire Sainte-Justine, Montreal, QC, Canada; Centre Hospitalier Universitaire Sainte-Justine, Montreal, QC, Canada

## Abstract

**Background:**

Although inflammatory bowel disease (IBD) severity is associated with decreased body mass index (BMI) in pediatric patients, many of them gain weight during follow-up. Physical activity levels (PAL) are associated with lower BMI dynamics while overweight/obesity worsen health outcomes in children. Few studies have explored BMI evolution and its related factors in this population.

**Aims:**

To investigate BMI dynamics in children with IBD from diagnosis to last follow-up and assess the impact of PAL, disease phenotype/activity, and medication on BMI changes.

**Methods:**

Pediatric IBD patients prospectively completed the Canadian Health Measure Survey quarterly for one year to assess PAL and were classified into three groups based on PAL (sedentary, moderately active or extremely/vigorously active). Clinical disease activity was classified using physician global assessment, PUCAI and sPCDAI. BMI was assessed at each visit.

**Results:**

A total of 256 patients (56% male, median age 14y [IQR (interquartile range): 13-16]) were included. At diagnosis, 56% were normal weight, 38% underweight, 5% overweight, and 2% obese. By the first visit, 74% were normal weight, 4% underweight, 17% overweight, and 5% obese. The median time from diagnosis to the first study visit was 29 months [IQR: 7-44] during which patients gained a mean of 2.97 (95% CI: 2.64;3.31) BMI points.

The following factors were associated with BMI gain: disease duration (Pearson Coefficient Correlation [95% CI]:0.42 [0.31;0.51]), age at diagnosis (-0.13 [-0.25; -0.01]), BMI at diagnosis (-0.23 [-0.34; -0.11]), BMI z-scores at diagnosis (-0.32 [-0.42; -0.20]) and higher age at inclusion (0.20 [0.08;0.31]).

In a multivariate logistic regression, we found that being underweight compared to normal weight (adjusted odds ratio (aOR) =0.15 [0.06; 0.39]) and exposition to anti-TNFα (aOR = (3.89 [1.17; 12.97]) were independently associated to the risk for overweight/obesity during follow-up.

**Conclusions:**

We observed a 15% increase in overweight/obesity prevalence and a 34% decrease in underweight prevalence in patients over the studied interval. BMI gain was significantly linked to exposure to anti-TNFa, warranting further investigations.

Table 1. Mean BMI Differences Between Diagnosis and First Study Visit

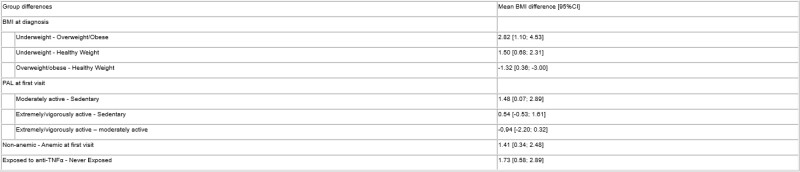

**Funding Agencies:**

None

